# Annotations

**Published:** 1897-09-25

**Authors:** 


### ANNOTATIONS.

### Board Schools and Fevers.

WE drew attention last week to the fact that, notwithstanding all the money this country spends on education, it still hesitates to pay for skilled inspection of the children in the schools, with the object of weeding out early cases of infectious disease. Curiously enough, last week a letter from the Clerk to the London School Board was published in *London*, which shows that not only do our school boards decline to provide such examination, but refuse, not altogether in the most civil terms, to accept it when it is offered to them. It appears that a dozen cases of scarlet fever occurred in a certain group of streets, and that Dr. Sidney Davies, the medical officer of health, being anxious to stamp out the disease, visited a school in the immediate neighbourhood, and having obtained the permission of the head teacher of infants to examine the children, discovered among 30 examined, three who he thought might be the conveyors of infection. When the 30 had been examined the mistress decided that she had better communicate with the Board before going any further, and asked him to desist, which he did. All this, of course, was reasonable enough. The pigheadedness comes up in the official letter which was straightway sent from the School Board to the vestry protesting against the action of their medical officer. It ends as follows: "The Board wish me to inform your vestry that they consider the action of Dr. Davies altogether unwarrantable and unjustifiable, and that the Board cannot allow him or any other medical officer to hold such an examination in any of their schools, or to allow their teachers to permit such examination to be made." Can dog-in-the-manger stupidity go further?

### Kneippism.

ATTENTION has lately been directed afresh to that curious compound of faith cure and hydropathy which is associated with the name of the late Father Kneipp. Few things are more curious in the history of medicine, taking medicine for the nonce to include all the quackeries, as in fact it did in former times, than to note the small relation that seems often to have existed between the amount of knowledge possessed by some of its professors and the sway they have been able to exercise over their followers. The study of quacks, and of the extraordinary forms which their quackery has taken, is in fact one of no small interest, for strange and grotesque as have been the means of cure which they have inflicted on their confiding patients, the men themselves have often been of striking personality. Father Kneipp seems to have been such a man, and we can well believe that in addition to the tonic effects of the regimen he ordered, tonic that is to those who could bear it, his own personal influence had much to do with the results which seemed to follow his "cure" in certain neurotic cases. It is difficult to speak seriously of Kneippism. There is a something comic about the whole performance which lends itself to ridicule. Nevertheless, we can have no doubt that some people did get benefit who had "stuck fast," if we may use the expression, under more ordinary forms of treatment. That Father Kneipp was honest we may well believe. But that he wallowed in ignorance up to the end is shown by the book he published shortly before his death. What he taught, then, goes for nothing; but he did hit upon a line of treatment which turned out to be beneficial to some whose sufferings were the result of coddling. The fact that many people lived through his treatment, and that some were the better for it, is enough to show that, from mere habit and from liking to be comfortable, we many of us coddle far too much and wear too many clothes. Let us admit that he did show that, and demonstrated it in a way that had not occurred to others. But it is not necessary now to go to Worishofen to discover it, nor, indeed, to apply the knowledge.

### Co operative Celibacy.

A STATEMENT of considerable interest in regard to the housing of the poor was lately made by Dr. Collins, chairman of the London County Council. This was to the effect that the year's working of the Council's model lodging-house in Drury Lane at a charge of sixpence per bed per night had resulted in a considerable profit. If such be the case we seem to be within a reasonable distance of providing a decent and sanitary lodging for, say, fivepence a night, or a penny more than the ordinary doss-house. From a mere public health point of view this is all gain. Both to the individual and the community it is an advantage that the lowest stratum of society should be decently lodged, brought under sanitary influences, and subjected to continuous sanitary supervision. From an economic standpoint, however, there is another side to the question, which has not attracted that attention which it deserves. Hitherto life in common lodging-houses has been under a social ban, and moreover has been accompanied with such discomforts that it has been shunned except by those who have been forced to it by misfortune. A whole series of agencies, however, are now at work in taking away the stigma which has hitherto attached to these houses, and in rendering them in a sense attractive residences. What, then, will be the effect on wages, on morality, and on the population of an arrangement which offers such advantages to celibate life as are provided by the Rowton or the County Council houses? It is obvious that the offer of a clean and comfortable bed, with society, reading-rooms, and other comforts—practically a club life—for three and sixpence a week must tend to a very large increase in celibacy, with all its advantages and disadvantages, among the first being increased comfort so long as wages keep up, among the latter an ultimate lowering of wages in response to the increased cheapness of such co-operative living. We have often been told that municipal dwellings and municipal lodging-houses spell Socialism, and the same has been said times without number in regard to the more liberal administration of the Poor Law. To these forms of Socialism no one objects, so long as they affect only a small minority—the wreckage of our social system. It would be another matter, however, if such beneficent Socialism as is involved in the provision of clean lodgings for the very poor were found to work in the direction of lowering wages and rendering married life in towns increasingly difficult, if not impossible, to working people.

